# Increased oxygen uptake in well-trained runners during uphill high intensity running intervals: A randomized crossover testing

**DOI:** 10.3389/fphys.2023.1117314

**Published:** 2023-02-16

**Authors:** Steffen Held, Ludwig Rappelt, René Giesen, Tim Wiedenmann, Jan-Philip Deutsch, Pamela Wicker, Lars Donath

**Affiliations:** ^1^ Department of Intervention Research in Exercise Training, German Sport University Cologne, Cologne, Germany; ^2^ Department of Fitness and Health, IST University of Applied Sciences, Duesseldorf, Germany; ^3^ Department of Movement and Training Science, University of Wuppertal, Wuppertal, Germany; ^4^ Department of Sports Science, Bielefeld University, Bielefeld, Germany

**Keywords:** incline, intervals, performance, injury, running

## Abstract

The time spent above 90% of maximal oxygen uptake ( $\dot{V}$ O_2_max) during high-intensity interval training (HIIT) sessions is intended to be maximized to improve $\dot{V}$ O_2_max. Since uphill running serves as a promising means to increase metabolic cost, we compared even and moderately inclined running in terms of time ≥90% $\dot{V}$ O_2_max and its corresponding physiological surrogates. Seventeen well-trained runners (8 females & 9 males; 25.8 ± 6.8yrs; 1.75 ± 0.08m; 63.2 ± 8.4kg; $\dot{V}$ O_2_max: 63.3 ± 4.2 ml/min/kg) randomly completed both a horizontal (1% incline) and uphill (8% incline) HIIT protocol (4-times 5min, with 90s rest). Mean oxygen uptake ( $\dot{V}$ O_2_mean), peak oxygen uptake ( $\dot{V}$ O_2_peak), lactate, heart rate (HR), and perceived exertion (RPE) were measured. Uphill HIIT revealed higher (*p* ≤ 0.012; partial eta-squared (pes) ≥ 0.351) $\dot{V}$ O_2_mean (uphill: 3.3 ± 0.6 vs. horizontal: 3.2 ± 0.5 L/min; standardized mean difference (SMD) = 0.15), $\dot{V}$ O_2_peak (uphill: 4.0 ± 0.7 vs. horizontal: 3.8 ± 0.7 L/min; SMD = 0.19), and accumulated time ≥90% $\dot{V}$ O_2_max (uphill: 9.1 ± 4.6 vs. horizontal: 6.4 ± 4.0 min; SMD = 0.62) compared to even HIIT. Lactate, HR, and RPE responses did not show mode*time rANOVA interaction effects (*p* ≥ 0.097; pes ≤0.14). Compared to horizontal HIIT, moderate uphill HIIT revealed higher fractions of $\dot{V}$ O_2_max at comparable perceived efforts, heartrate and lactate response. Therefore, moderate uphill HiiT notably increased time spent above 90% $\dot{V}$ O_2_max.

## 1 Introduction

High level endurance training requires large training volumes (Seiler, 2010). In elite athletes, commonly, a high proportion of this training volume is performed at low training intensities (Seiler, 2010). However, to achieve an optimal metabolic training stimulus on maximal oxygen uptake ( $\dot{V}$ O_2_max), it has been recommended to perform a certain amount of high-intensity interval training (HIIT). This recommendation is especially relevant for well-trained endurance athletes (Laursen and Jenkins, 2002). Thereby, HIIT involves repeated bouts of high-intensity exercise interspersed with recovery periods (Laursen and Jenkins, 2002; Buchheit and Laursen, 2013). This training method mainly focuses on $\dot{V}$ O_2_max improvements (Midgley et al., 2006; Buchheit and Laursen, 2013), as the upper limit to the aerobic metabolism and a key determinant of endurance performance (Joyner and Coyle, 2008). In order to improve $\dot{V}$ O_2_max in highly trained endurance athletes, it has been suggested that a prolonged time at intensities corresponding to a high percentage of maximal oxygen uptake is important (Wenger and Bell, 1986; Midgley et al., 2006). Therefore, the quality of a HIIT session can be defined by mean oxygen uptake ( $\dot{V}$ O_2_mean) or accumulated training time ≥90% $\dot{V}$ O_2_max (Midgley et al., 2006; Turnes et al., 2016). This adaptational potential has been attributed to the large metabolic stimulus for myocardial morphological adaptations that increases maximal cardiac stroke volume and also increased peripheral skeletal muscle adaptations (Midgley et al., 2006).

In both prospective and cohort studies, a high weekly running volume has been associated with running-related injuries (Macera et al., 1989; Walter et al., 1989). Although the causes of running injuries are multifactorial, in this context, the runner’s interaction with the ground and the resulting reaction force has been considered to be one risk factor (Zadpoor and Nikooyan, 2011; Daoud et al., 2012). Thus, higher loading rates were associated with increased risk of sustaining an injury (Crowell and Davis, 2011; Futrell et al., 2018). More recently, however, in a prospective case control-study in recreational runners, the vertical impact peak and loading rate were not associated with a higher injury rate (Malisoux et al., 2022). Furthermore, in collegiate cross country runners, an higher occurrence rate of bone stress injuries has been linked to a higher step rate, but not higher ground reaction forces (Kliethermes et al., 2021). Nevertheless, besides adequate periodization and polarization models in endurance sports, reducing loading rates is still recommended as an effective means to reduce the risk of developing running injuries (Bowser et al., 2018). In this context, increasing the slope might lead to a significantly lower vertical loading rate during uphill running compared to flat level running (Gottschall and Kram, 2005; Lemire et al., 2022a). Also, increasing the slope from flat level running to 7% was found to reduce flight time and increase floor contact time, in turn resulting in highly significant increases in step frequency (Padulo et al., 2013). Apart from this, previous research revealed an increased energy cost *via* uphill running compared to horizontal running (Lemire et al., 2022b). Additionally, when running at the same velocity, uphill running is more metabolically demanding than horizontal running (Minetti et al., 2002; Vernillo et al., 2017), hence allowing a similar training stimulus at a lower running velocity.

Against this background, this randomized crossover testing examined the peak $\dot{V}$ O_2_, mean $\dot{V}$ O_2_ and accumulated time spent ≥90% $\dot{V}$ O_2_max during moderate slope uphill compared to horizontal HIIT running. We assumed similar $\dot{V}$ O_2_ data and reduced running speed during uphill HIIT. The findings of the present study might be impactful for designing and integrating HIIT session within polarization models and in terms of training variations to minimize injury risks in runners with high training volumes.

## 2 Materials and methods

### 2.1 Participants

G*Power (Version 3.1.9.6) was employed to perform an *a priori* power analysis. Based on increased metabolic costs *via* uphill running (Minetti et al., 1994; 2002; Vernillo et al., 2017) moderate effect sizes (standard mean differences (SMD) = 0.60) between horizontal and uphill HIIT running were assumed. A sample size of n = 13 was determined, using the following statistical indicators (*α* = 0.05; study power (1-β-error) = 0.95; one tail). Assuming moderate dropouts (15%–20%), n = 17 well-trained runners were enrolled in this acute randomized controlled crossover testing. These participants consisted of 8 female (age: 24.4 ± 3.7 yrs; height: 1.69 ± 0.07 m; body mass: 56.6 ± 5.8 kg; body fat: 14.6 ± 4.8%; $\dot{V}$ O_2_max: 60.5 ± 2.3 ml/min/kg; running volume: 58.1 ± 18.5 km/week) and 9 male (age: 27.1 ± 8.8 yrs; height: 1.80 ± 0.07 m; body mass: 69.1 ± 5.6 kg; body fat: 9.7 ± 3.1%; $\dot{V}$ O_2_max: 65.7 ± 4.1 ml/min/kg; running volume: 65.0 ± 20.3 km/week) trained runners. Inclusion criteria were running experience of at least 3 years, running volume of at least 40 km/week, and no medical condition that potentially impedes the completion of testing and training. The study was approved by the local ethical committee (153/2022), fulfilled the international ethical standards, and all participants signed an informed written consent prior to the start of the study.

### 2.2 Testing procedures

The measurements were conducted within four lab visits over 3 weeks for each participant. Thereby, horizontal and uphill $\dot{V}$ O_2_max tests (lab visit 1 & 2) as well as horizontal and uphill HIIT protocols (lab visit 3 & 4) were performed. Adapted from previous research (Rønnestad et al., 2019; 2022), the HIIT protocol consisted of four 5-min intervals with 90 s passive rest in between. During HIIT sessions, participants were instructed to run at their maximal sustainable intensity during all four interval bouts (*isoeffort*) (Seiler and Hetlelid, 2005). Therefore, participants could increase or decrease the velocity individually. All measurements were conducted on a motorized treadmill (PPS Med treadmill, Woodway, Waukesha, USA), with the horizontal conditions being performed at 1% incline and the uphill conditions being performed at 8% incline. To avoid sequencing effects, the first two and the last two lab visits were individually performed in a randomized order. At least 96 h rest was ensured between each lab visit. Participants were further instructed to avoid any strenuous exercise 2 days before each testing session. To control for potential circadian effects on performance, all measurements were conducted at similar day times for each participant. A standardized 15-min warm-up (easy running, including knee lift, heel lift, external rotation hip, internal rotation hip, 10 lunges alternating, 10 squats, individual dynamic stretching) was performed prior to each lab session.

Spirometric data during all lab visits were collected using a breath-by-breath system (Zan 600 Oxi USB, Zan Messgeräte, Oberthulba, Germany). This spirometric system was calibrated prior to each test, following the manufacturer’s recommendations. To determine uphill and horizontal-running $\dot{V}$ O_2_max, an incremental ramp testing protocol was performed at horizontal (1% incline) and uphill (8% incline) conditions (lab visit 1 & 2). Adapted from previous research with similar $\dot{V}$ O_2_max values (Baumgartner et al., 2021), the initial velocity for both ramp tests was set based on prior running experience and estimated 10 km race time for each participant individually at 2, 2.5, or 3 m/s. The ramp protocol then consisted of 0.2% increases every 30 s until the participant reached exhaustion (Midgley et al., 2007). All participants were verbally encouraged and motivated in the same way towards the end of each test. The highest consecutive oxygen uptake values within 30 s during the final part of the ramp tests were considered as $\dot{V}$ O_2_max. For both conditions, $\dot{V}$ O_2_max and objective exhaustion were verified for each participant following the corresponding criteria (Midgley et al., 2007). All participants fulfilled these objective exhaustion criteria (i.e., at least 4 out of 6 criteria). Adapted from previous research, the quality of both HIIT sessions were defined by mean $\dot{V}$ O_2_ and accumulated training time ≥90% $\dot{V}$ O_2_max (Time90) (Midgley et al., 2006; Thevenet et al., 2007; Turnes et al., 2016). Since both HIIT sessions were time matched with the same work to rest ratio, mean $\dot{V}$ O_2_ and Time90 were determined based on the entire training session (interval with pauses). Furthermore, to determine Time90, the entire training session (interval with pauses) was normalized to seconds, subsequently seconds with $\dot{V}$ O_2_ value ≥ $\dot{V}$ O_2_max were summed up. Thereby, the highest $\dot{V}$ O_2_max value of the horizontal or incline ramp test was used as reference values. Furthermore, peak oxygen consumption (highest oxygen uptake during the intervals averaged over 30 s; $\dot{V}$ O_2_peak) during both HIIT protocols was additionally considered. Apart from this, total respiration per minute (minute volume), respiratory frequency (breath frequency), and tidal volume were also used for further data analysis. In addition, capillary blood samples were taken from the earlobe of the participants for lactate analysis (EBIOplus; EKF Diagnostic Sales, Magdeburg, Germany), heart rate (HR) was measured using a heart rate strap (Polar, Kempele, Finland), and perceived exertion levels were assessed based on RPE (CR-10 scale) (Foster et al., 2001) prior to the first interval and immediately after each running interval.

### 2.3 Statistics

Data are presented as means ± standard deviations. Normal distribution was initially tested using Shapiro-Wilk tests (*p* ≥ 0.1). Variance homogeneity was visually confirmed *via* plotting sampled residuals vs. theoretical (ideal) residuals (Kozak and Piepho, 2018). Sphericity was verified *via* Mauchly´s tests. To examine mode differences (horizontal vs. uphill) for the respective outcome measures ( $\dot{V}$ O_2_, $\dot{V}$ O_2_peak, $\dot{V}$ O_2_max, Time 90, minute volume, breath frequency, and tidal volume), numerous separate two-way (mode: horizontal vs. uphill) repeated measurement analysis of variances (rANOVA) were conducted. 2 (mode: horizontal vs. uphill) × 4 (time: pre vs. interval 1 vs. interval 2 vs. interval 3 vs. interval 4) rANOVAs were calculated for lactate, HR, and RPE, and running velocity data. rANOVA effect sizes are given as partial eta squared (pes) with ≥0.01, ≥0.06, and ≥0.14 indicating small, moderate, and large effects, respectively (Cohen, 1988). In case of significant mode × time interaction effects, Bonferroni *post hoc* tests were subsequently computed. For pairwise effect size comparison, standard mean differences (SMD) were additionally calculated as the differences between means divided by the pooled standard deviations (trivial: SMD <0.2; small: 0.2 ≤ SMD <0.5; moderate: 0.5 ≤ SMD <0.8; large SMD ≥0.8) (Cohen, 1988). Furthermore, the smallest worthwhile change was calculated as 30% of baseline standard deviation (Hopkins, 2004). Pearson correlation coefficients were calculated in order to define the relationships of the measured variables. A correlation coefficient of | r | ≈ 0.30 is interpreted as low/weak correlation, | r | ≈ 0.50 is interpreted as mean/moderate correlation and | r | ≈ 0.80 is interpreted as large/strong correlation (Cohen, 1988). Statistical analyses were conducted using R (version 4.0.5) and RStudio (version 1.4.1106) software.

## 3 Results

### 3.1 Incremental ramp test

No significant differences (*p* = 0.100; pes = 0.100; mean difference (MD) = 0.2 ± 0.5 L/min; SMD = 0.28) were found between horizontal (3.9 ± 0.7 L/min) and uphill $\dot{V}$ O_2_max (4.1 ± 0.7 L/min) during the incremental ramp tests.

### 3.2 HIIT sessions

**FIGURE 1:**
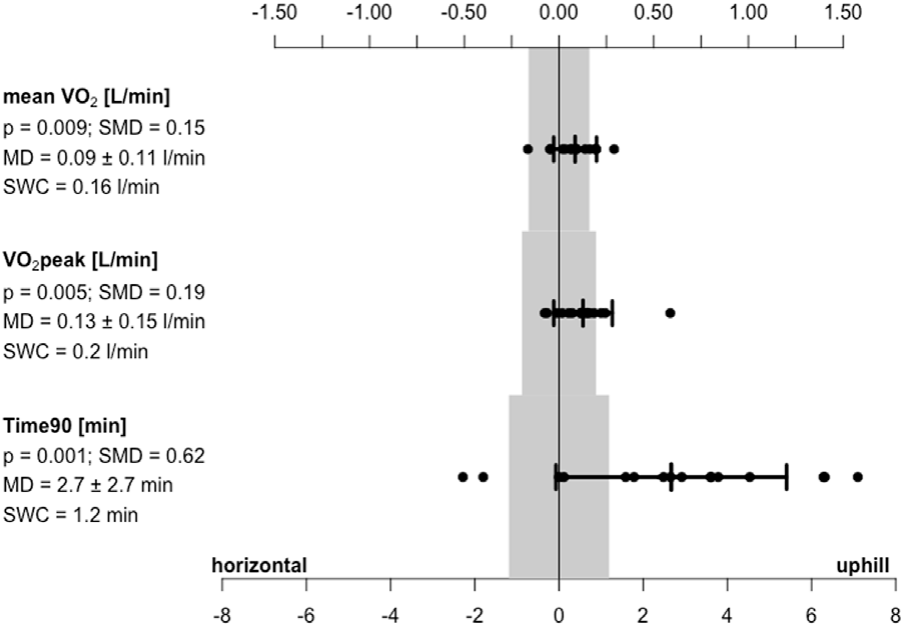
Mean difference (MD ± standard deviation) between horizontal and uphill high intensity training protocols for mean oxygen consumption ( $\dot{V}$ O_2_), peak oxygen consumption ( $\dot{V}$ O_2_peak), and accumulated time above 90% of maximal oxygen consumption (Time90). Smallest worthwhile change (SWC) boundaries are marked in grey. Significance levels (*p*) and pairwise effect sizes as standard mean differences (SMD) are presented.

rANOVA revealed significant effects (*p* ≤ 0.012; pes ≥0.351) regarding $\dot{V}$ O_2_, $\dot{V}$ O_2_peak, Time90, minute volume, breath frequency, and tidal volume (Figure 1). Thereby, uphill HIIT showed higher values than horizontal HIIT for $\dot{V}$ O_2_mean (3.3 ± 0.6 vs. 3.2 ± 0.5 L/min; MD = 0.1 ± 0.1 L/min; SMD = 0.15), $\dot{V}$ O_2_peak (4.0 ± 0.7 vs. 3.8 ± 0.7 L/min; MD = 0.1 ± 0.2 L/min; SMD = 0.19), Time90 (9.1 ± 4.6 vs. 6.4 ± 4.0 min; MD = 2.7 ± 2.7 L/min; SMD = 0.62), and tidal volume (2144 ± 511 vs. 2061 ± 502 ml; MD = 83 ± 117 ml; SMD = 0.16). In contrast, uphill HIIT revealed lower values than horizontal HIIT for minute volume (94.3 ± 15.1 vs. 101.2 ± 17.3 L/min; MD = 6.9 ± 8.4 L/min; SMD = 0.43) and breath frequency (44.9 ± 6.0 vs. 50.5 ± 9.2 breaths/min, MD = 5.6 ± 5.9 breaths/min; SMD = 0.73). Furthermore, only for Time90, breath frequency and minute volume, the differences between conditions exceeded the smallest worthwhile change. Furthermore, Time90 revealed high (r = 0.82) and significant (*p* < 0.001) correlations between horizontal and uphill HIIT.

**FIGURE 2:**
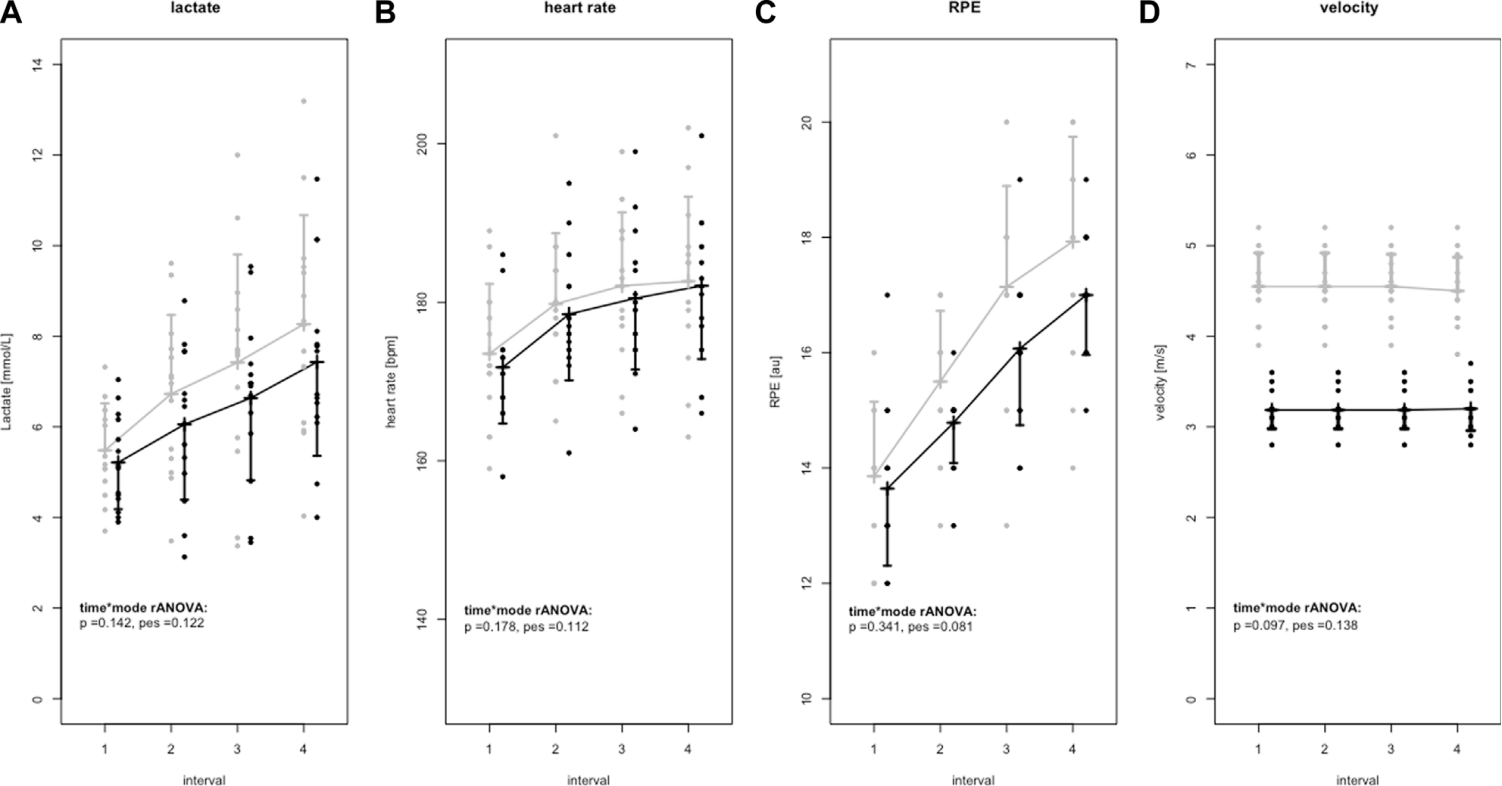
Lactate **(A)**, heart rate **(B)**, RPE **(C)**, and running velocity **(D)** data (mean ± standard deviation) of horizontal (grey) and uphill (black) high intensity training protocols. Individual values are marked as points. In addition, *p*-values of time*mode interaction effects (*p*) of the repeated measurement variance analyses (rANOVA) and corresponding effect sizes as partial eta squared (pes) are given.

No significant mode × time rANOVA interaction effects (*p* ≥ 0.097; pes ≤0.14) for lactate, HR, RPE and running velocity were found (Figure 2). Nevertheless, running velocity revealed significant time effects (*p* ≤ 0.001). Subsequently performed *post hoc* tests (*p* ≤ 0.001; SMD ≥3.53) revealed higher running velocity during horizontal HIIT (4.47 ± 0.33 to 4.51 ± 0.35 m/s) compared to uphill HIIT (3.17 ± 0.18 to 3.18 ± 0.21 m/s) during all intervals.

## 4 Discussion

To the best of our knowledge, this is the first acute randomized controlled crossover study that examined $\dot{V}$ O_2_, lactate, HR, and RPE response of time- and effort-matched horizontal vs. uphill HIIT running in well-trained runners. Our key findings were increased mean $\dot{V}$ O_2_, $\dot{V}$ O_2_peak, and accumulated training time ≥90% $\dot{V}$ O_2_max *via* uphill HIIT compared to horizontal HIIT. In contrast, lactate, HR, and RPE revealed no significant differences between horizontal and uphill HIIT protocols. Furthermore, horizontal and uphill ramp tests yielded similar $\dot{V}$ O_2_max values.

A higher acute oxygen consumption during uphill running is commonly explained by the fact that the use of elastic energy may be compromised, so that in turn more mechanical energy (i.e., greater concentric muscle activity) needs to be generated, in order to lift the body’s center of gravity upward and subsequently overcome the slope (Snyder and Farley, 2011). Thus, in the present study, uphill running during a HIIT session notably increased the mean time ≥90% $\dot{V}$ O_2_max by about 42%. Interestingly, this percentage increase is quite similar to previous cycling-related research, which used power-output variation within the work intervals (Bossi et al., 2020). In this previous study, two different interval training sessions, matched for duration and mean power output (6 × 5 min at a mean intensity of 84% of maximal aerobic power (MAP), with 2.5 min of rest between intervals), were performed. By performing several 30s bouts at 100% MAP within these intervals to increase the power-output variation within the work intervals, the mean time ≥90% $\dot{V}$ O_2_max increased by about 43% (Bossi et al., 2020). It thus seems that variation of the power-output by performing short bouts of sprinting or by employing inclination might be an important factor to increase the time ≥90% $\dot{V}$ O_2_max during HIIT sessions. In addition, and in line with our findings, lactate, HR, and RPE data reported by Bossi and colleagues (Bossi et al., 2020) were similar for both interval training conditions. However, both studies only focused on short-term effects. Therefore, Bossi and colleagues (Bossi et al., 2020) emphasized the need for longitudinal studies while speculating that performance adaptations will most likely be superior to constant-intensity work intervals. Based on our data, a 6-week period of uphill HIIT (2 sessions per week) would result in about half an hour more accumulated time ≥90% $\dot{V}$ O_2_max compared to horizontal HIIT. This additional accumulated time ≥90% $\dot{V}$ O_2_max *via* uphill HIIT is equivalent to 5 horizontal HIIT sessions. Therefore, superior performance adaptations could be assumed *via* uphill HIIT. This assumption is supported by increased $\dot{V}$ O_2_max and power output at the lactate threshold adaptations over a 4-week training period, if recreationally-trained cyclists spent about 100s more time above 90% $\dot{V}$ O_2_max per training session (Turnes et al., 2016). In line with these findings, the accumulated training time ≥90% $\dot{V}$ O_2_max is frequently considered a highly important marker for efficient HIIT sessions designed to increase $\dot{V}$ O_2_max (Midgley et al., 2006; Thevenet et al., 2007; Turnes et al., 2016). Our findings of HIIT protocols performed at the maximal sustainable intensity during all four interval bouts (*isoeffort*) (Seiler and Hetlelid, 2005) revealed increased mean $\dot{V}$ O_2_, $\dot{V}$ O_2_peak, and accumulated time above 90% $\dot{V}$ O_2_max at a decreased running velocity during the uphill HIIT condition and similar lactate, HR, and RPE values. However, as at a given speed, uphill running results in higher $\dot{V}$ O_2_, lactate, HR, and RPE data compared to horizontal running (Minetti et al., 1994; 2002; Vernillo et al., 2017), it might be possible that the maximum oxygen uptake differs between running uphill compared to level running conditions. Nevertheless, we did not find significant differences in $\dot{V}$ O_2_max in the initial incremental ramp tests performed at horizontal running condition and 8% slope. This is in line with results reported by Lemire and colleagues (Lemire et al., 2020) who reported similar $\dot{V}$ O_2_max values in well-trained trail runners performing step tests on a treadmill in level and 15% uphill running conditions. However, a different study conducted in well-trained trail runners comparing the physiological responses to step tests with increasing gradient reported significantly higher $\dot{V}$ O_2_max values at gradients of 40% compared to level running (Cassirame et al., 2022). This has also been described by Margaria and colleagues (Margaria et al., 1963): According to their work, when running on positive gradients up to 15% incline the minimum energy cost of running increases as a function of the incline. At slopes above 20%, however, the energy cost becomes equal to that of concentric muscular work (Minetti et al., 2002). It therefore seems, that at least in special populations (i.e., trail runners) and at very steep inclination (i.e., above 15%) the maximal oxygen uptake might significantly and relevantly differ from level running. Hence, this should be taken into account, when quantifying training load as a percentage value of the maximal oxygen uptake.

Previous research revealed that 19%–79% of runners report musculoskeletal injuries of the lower extremities annually (van Gent et al., 2007). Thereby, loading rate and ground reaction force were repeatedly named as relevant risk factors (Crowell and Davis, 2011; Zadpoor and Nikooyan, 2011; Futrell et al., 2018). These relationships, however, were often established based on retrospective, cross-sectional data. More recently, in prospective case control-studies comprising recreational (Malisoux et al., 2022) and collegiate cross country runners (Kliethermes et al., 2021), the vertical impact peak and loading rate were not associated with a higher injury rate. Nevertheless, reducing loading rates is still recommended as an effective means to reduce the risk of developing running injuries (Bowser et al., 2018). In this context, uphill running revealed decreased ground reaction force data compared to horizontal running (Gottschall and Kram, 2005). Furthermore, we observed decreased running velocities during uphill HIIT compared to horizontal HIIT, which additionally decrease loading rate and ground reaction force (Keller et al., 1996). In detail, previous research revealed a 22%–39% ground reaction force decrease *via* an 6%–9% slope increase (Gottschall and Kram, 2005; Kowalski and Li, 2016). Furthermore, slower running resulted in reduced ground reaction force (Keller et al., 1996). Based on our running velocity differences between horizontal and uphill HIIT, this would result in a ground reaction force reduction of 11%. For the present study a possible reduction of loading rates remains, however, speculative, as these loading rates and ground reaction forces were not measured. Thus, more adequately powered prospective studies are necessary to investigate the association of musculoskeletal injuries of the lower extremities and loading rate as well as the potential prevention effect of uphill running.

Horizontal running has been linked to the stretch-shortening cycle of the muscle-tendon unit of the lower limb (Schöffl et al., 2021), in which part of the mechanical energy of the center of mass (COM) is absorbed during the negative work phase to be restored during the next positive work phase (Nicol et al., 2006). This storage and release of kinetic and potential energy contributes to the acceleration of the body upwards during the propulsive phase and to the reduction of the energy production needed during the concentric phase (Snyder and Farley, 2011; Snyder et al., 2012). In contrast, during uphill running, the center of mass needs to be propelled vertically and does not oscillate around an equilibrium (Dewolf et al., 2016). In detail, the center of mass loses horizontal while simultaneously gaining vertical velocity during the first part of ground contact. Subsequently, during the second part of the contact, a fraction of the energy stored in the elastic elements of the muscle tendon unit is released to increase the kinetic and potential of the center of mass (Dewolf et al., 2016). Accordingly, differences in muscle activation patterns of the lower extremities have been reported between horizontal and uphill running (Yokozawa et al., 2007), with concentric muscle work being dominant during uphill running (Giandolini et al., 2016). Furthermore, to increase the running velocity in flat running conditions, athletes tend to increase their stride length and frequency almost linearly (Ito et al., 1983; Cavanagh and Kram, 1989; Brisswalter and Legros, 1995). Simultaneously, the floor contact time and flight time are reduced (Ito et al., 1983; Cavanagh and Kram, 1989; Brisswalter and Legros, 1995). Even though this pattern is also visible during uphill running compared to flat running, stride length and flight time are significantly reduced, since the foot touches the belt or ground earlier (Padulo et al., 2012; 2013). As the floor contact time does not seem to differ between flat and uphill running, this subsequently leads to a significant reduction in flight time during the uphill running condition (Padulo et al., 2012; 2013). Therefore, it seems possible, that prolonged training sessions running uphill might change the athlete’s kinematics, thus resulting in a reduction in running economy at horizontal conditions. Nevertheless, at least for constant running velocities, experienced athletes select an individual combination of stride length and frequency resulting in the least energy cost (Cavanagh and Kram, 1989; Cavagna et al., 1991), while providing the greatest mechanical efficiency (Morgan et al., 1994). Even though only a small fraction of the overall training time is spent on high-intensity running (Stöggl and Sperlich, 2015), a potential longitudinal effect on running economy induced by prolonged uphill running should be addressed in further research.

A limitation that needs to be addressed is the lack of spatiotemporal running parameters including information on stride length and frequency. Thus, further research should try to disentangle the relationship between spatiotemporal running parameters and oxygen uptake during uphill running. In addition, the potential long-term training effects mentioned above should be examined in appropriate longitudinal intervention studies.

In conclusion, this randomized crossover testing revealed increased mean $\dot{V}$ O_2_, $\dot{V}$ O_2_peak, and accumulated training time ≥90% $\dot{V}$ O_2_max *via* uphill HIIT. Thus, uphill running during HIIT sessions appears to be an effective alternative to traditional horizontal HIIT sessions. Whether performance adaptations will be superior to horizontal running work intervals remains to be established by a longitudinal study, but similar lactate, HR, and RPE data suggest that it is unlikely that negative training outcomes occur. Nevertheless, future research should investigate whether training-induced adaptations can be improved *via* uphill HIIT. Furthermore, such further studies should also examine if different muscle activation patterns *via* uphill running (Giandolini et al., 2016) lead to adverse effects in terms of (horizontal) running economy.

## Data Availability

The raw data supporting the conclusions of this article will be made available by the authors, without undue reservation.
